# Tomentodione M sensitizes multidrug resistant cancer cells by decreasing P-glycoprotein via inhibition of p38 MAPK signaling

**DOI:** 10.18632/oncotarget.21949

**Published:** 2017-10-19

**Authors:** Xu-Wei Zhou, Yuan-Zheng Xia, Ya-Long Zhang, Jian-Guang Luo, Chao Han, Hao Zhang, Chao Zhang, Lei Yang, Ling-Yi Kong

**Affiliations:** ^1^ Jiangsu Key Laboratory of Bioactive Natural Product Research and State Key Laboratory of Natural Medicines, China Pharmaceutical University, Nanjing 210009, China

**Keywords:** tomentodione M, meroterpenoid, multidrug resistance, P-glycoprotein, p38

## Abstract

In this study, we investigated the mechanism by which tomentodione M (TTM), a novel natural syncarpic acid-conjugated monoterpene, reversed multi-drug resistance (MDR) in cancer cells. TTM increased the cytotoxicity of chemotherapeutic drugs such as docetaxel and doxorubicin in MCF-7/MDR and K562/MDR cells in a dose- and time-dependent manner. TTM reduced colony formation and enhanced apoptosis in docetaxel-treated MCF-7/MDR and K562/MDR cells, and it enhanced intracellular accumulation of doxorubicin and rhodamine 123 in MDR cancer cells by reducing drug efflux mediated by P-gp. TTM decreased expression of both P-gp mRNA and protein by inhibiting p38 MAPK signaling. Similarly, the p38 MAPK inhibitor SB203580 reversed MDR in cancer cells by decreasing P-gp expression. Conversely, p38 MAPK-overexpressing MCF-7 and K562 cells showed higher P-gp expression than controls. These observations indicate that TTM reverses MDR in cancer cells by decreasing P-gp expression via p38 MAPK inhibition.

## INTRODUCTION

Multidrug resistance (MDR) is the phenomenon by which neoplastic cells display resistance to many chemotherapeutic drugs that are chemically dissimilar with different cytotoxic targets [1]. MDR negates the efficacy of cancer chemotherapy and results in therapeutic failure, which is detrimental for patient survival. Hence, MDR is the biggest challenge for strong and effective cancer treatment.

Currently, numerous chemotherapeutics are used in the clinic for cancer treatment such as doxorubicin (DOX), Daunorubicin (DNR), Epirubicin (EPI), Docetaxel (Doc) and Cisplatin (DDP) [2]. However, intrinsic or acquired drug resistance such as MDR leads to failure of chemotherapy. In most cases, chemotherapy becomes ineffective over time because of resistance. Moreover, the acquired resistance of the neoplastic cells is not limited to the original drug, but, also affects a wide variety of structurally and mechanistically unrelated drugs. Therefore, new therapeutic strategies are needed to overcome chemotherapy resistance in MDR cancer cells.

The mechanisms underlying MDR in cancer cells are complicated and not completely understood. Some of the MDR-related mechanisms include decreased drug accumulation, alterations in cell cycle checkpoints, resistance to cellular apoptotic mechanisms and repair of damaged cellular targets [3]. The most common and well studied mechanism of MDR in cancer cells is over-expression of ATP-binding cassette (ABC) transporters, whose regulation is highly complex and results in decreased drug accumulation [4, 5]. The most extensively studied MDR transporters include ABCB1 (also known as MDR1 or P-gp), ABCC1 (also known as MRP1) and ABCG2 (also known as BCRP or MXR). ABCA1 was originally found as the primary gatekeeper for eliminating cholesterol that played an important role in protecting against cardiovascular disease [6]. It has also been implicated in multi-drug resistance apart from its role in reverse cholesterol metabolism [7, 8]. Moreover, many groups have reported P-gp as a target for antitumor therapy in drug-resistant tumors [9–11].

To date, P-gp is the best studied among the ABC-transporter-mediated MDR proteins [12–14]. The hallmark of chemo-resistance in cancer cells is P-gp overexpression. High P-gp levels are associated with poor survival rate in osteosarcoma [15], lymphomas [16], small-cell lung cancer [17], breast cancer [18] and leukemias [19]. Therefore, P-gp inhibitors are necessary to treat cancer patients that demonstrate MDR. Many P-gp inhibitors such as Valspoder (PSC-833), dofequidar fumarate (MS-209), thiosemicarbazone derivative (NSC73306) Zosuduidar (LY335979) and Tariquidar (XR9567) have been found to antagonize P-gp function [20–23]. However, phase III trials of these drugs have been disappointing, primarily because of low host tolerance to these experimental MDR modulators that precludes the attainment of active intracellular levels [24, 25]. Moreover, these drugs demonstrate adverse effects in patients and alter the pharmacokinetics of co-administered anticancer drug [26]. Also, none of these inhibitors have achieved significant survival benefits [27]. Therefore, there is an urgent need for novel MDR antagonists that demonstrate low toxicity and high efficacy.

**Figure 1:**
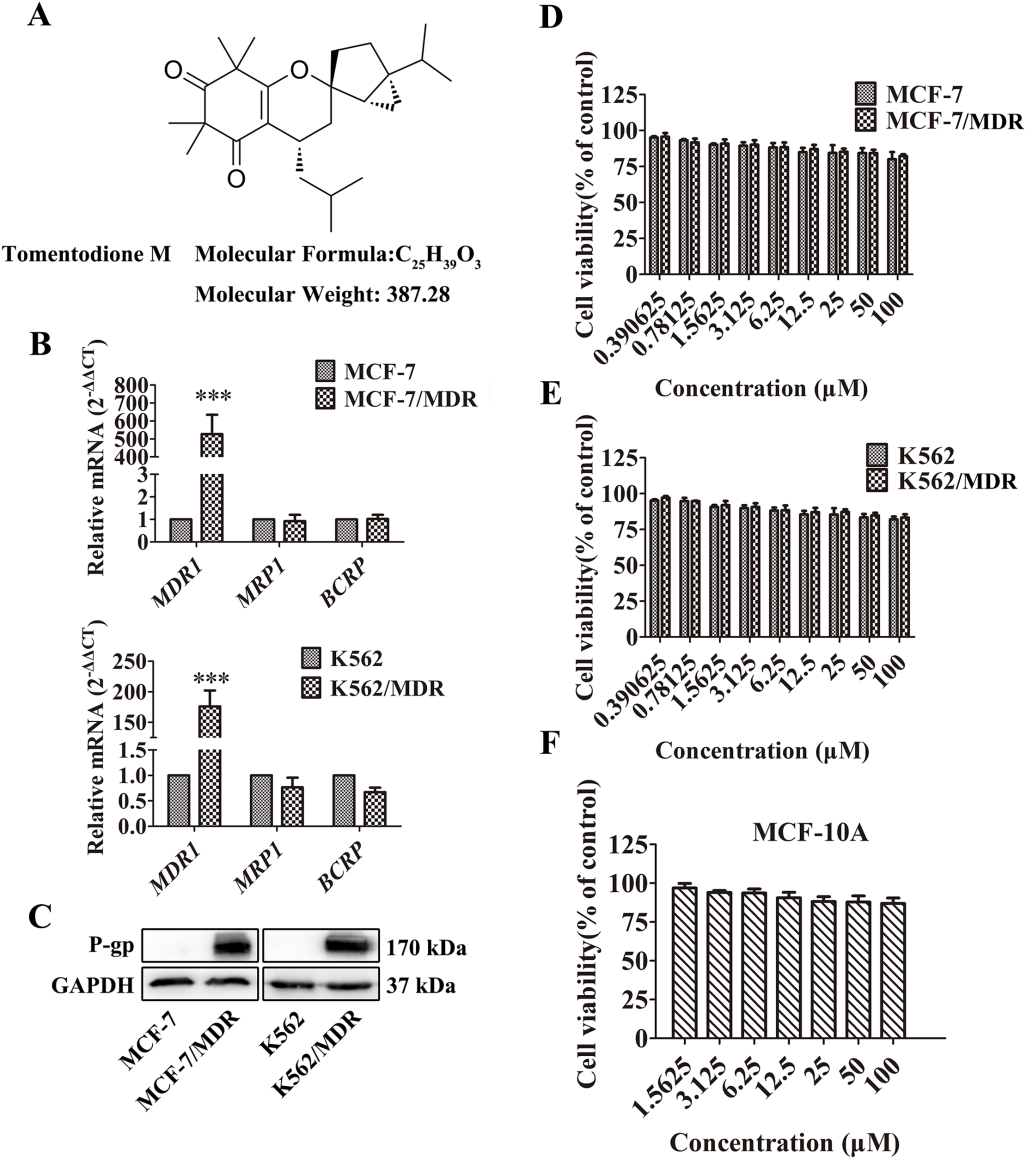
Effect of TTM on cancer cell toxicity and expression of ABC transporters **(A)** Chemical structure and molecular weight of Tomentodione M (TTM). **(B)** QRT-PCR analysis of *MDR1*, *MRP1* and *BCRP* mRNA levels relative to GAPDH in parental and multi-drug resistant (MDR) MCF-7 and K562 cancer cells. Note: The relative mRNA levels are expressed as fold-changes relative to control group, which is arbitrarily represented as 1. The data are representative of at least 6 replicates. **(C)** Representative western blot showing P-gp protein levels in MCF-7/MDR and K562/MDR cells relative to their corresponding parental cells. GAPDH was used as loading control. **(D-F)** MTT and CCK-8 assays showing cell viability of multidrug resistant cell lines (MCF-7/MDR and K562/MDR) and their corresponding parental cell lines (MCF-7 and K562), as well as non-tumor cell line (MCF-10A) treated with 0-100 μM TTM for 48 hr. Inhibition of cell proliferation by different concentrations of TTM were calculated based on the ratio of absorbance in treatment and control samples. The absorbance was evaluated at a test wavelength of 570 nm, and a reference wavelength of 630 nm in MTT assays. The absorbance at 450 nm was used in CCK-8 assays. Note: Values represent mean ± SEM from three independent experiments. ^***^ denotes *P* < 0.001 compared to control.

Traditional Chinese Medicine (TCM) has offered many alternative natural products that demonstrate great potential as MDR inhibitors with low adverse effects. These natural products decrease P-gp expression [21, 28, 29]. Recent studies have shown that natural terpenoids reverse MDR in cancer cells [30, 31]. However, their mechanism of action is unknown. Therefore, in this study, we investigated the mechanism of action of a novel meroterpenoid, tomentodione M (TTM) (Figure 1A) in reversing MDR in MCF-7 and K562 cancer cells.

## RESULTS

### MDR cancer cells overexpress P-gp

We first evaluated the cytotoxicity of several anticancer agents such as DOX, DNR, EPI, DDP and Doc in MDR and parental MCF-7 and K562 cell lines. As shown in Table 1, the MCF-7/MDR and K562/MDR cells showed higher IC_50_ for DOX, DNR, EPI and Doc than the parental MCF-7 and K562 cells, thereby demonstrating drug resistance. However, the IC_50_ values for DDP were similar in the parental and MDR MCF-7 and K562 cell lines. The resistance index for DOX, DNR, EPI and Doc was 16.60, 29.25, 29.94 and 22.55 in MCF-7/MDR cells, and 181, 72.85, 2664.65 and 7571.1 in K562/MDR cells (Table 1).

**Table 1:** Effect of TTM on cytotoxicity of chemotherapeutic drugs in parental and multi-drug resistant MCF-7 and K562 cells

Drug		IC_50_ ± SEM^a^ (μM) (reversal fold change (RF))^b^			
		MCF-7	MCF-7/MDR	K562	K562/MDR
Doxorubicin		0.632 ± 0.083 (1.00)	16.597 ±5.225 (1.00)	0.100 ± 0.005 (1.00)	18.104 ± 3.228 (1.00)
	+10 μM TTM	0.483 ± 0.088 (1.31)	6.939 ± 0.853 (2.39)	0.071 ± 0.018 (1.41)	5.364 ± 1.269 (3.38)
	+30 μM TTM	0.221 ± 0.024 (2.86)	2.286 ± 0.205 (7.26)^**^	0.039 ± 0.016 (2.56)	3.142 ± 1.115 (5.76)
	+10 μM Ver	0.346 ± 0.045 (1.83)	1.901 ± 0.350 (8.73)^***^	0.028 ± 0.009 (3.527)	0.414 ± 0.012 (43.73)^***^
Daunorubicin		0.114 ± 0.005 (1.00)	3.335 ± 0.646 (1.00)	0.048 ± 0.005 (1.00)	3.497 ± 0.405 (1.00)
	+10 μM TTM	0.113 ± 0.003 (1.01)	2.341 ± 0.199 (1.42)	0.045 ± 0.005 (1.07)	1.149 ± 0.239 (3.04)
	+30 μM TTM	0.109 ± 0.003 (1.05)	1.187 ± 0.105 (2.81)	0.040 ± 0.006 (1.20)	0.107 ± 0.012 (32.68)^***^
	+10 μM Ver	0.110 ± 0.004 (1.04)	0.423 ± 0.083 (7.88)^**^	0.037 ± 0.004 (1.28)	0.009 ± 0.001 (388.56)^***^
Epirubicin		0.217 ± 0.037 (1.00)	6.498 ± 1.606 (1.00)	0.034 ± 0.013 (1.00)	90.598 ± 12.804 (1.00)
	+10 μM TTM	0.193 ± 0.035 (1.12)	3.667 ± 0.198 (1.77)	0.022 ± 0.018 (1.54)	7.566 ± 1.040 (11.97)^***^
	+30 μM TTM	0.132 ± 0.029 (1.64)	1.766 ± 0.236 (3.68)	0.011 ± 0.025 (3.09)	1.919 ± 0.077 (47.21)^***^
	+10 μM Ver	0.125 ± 0.007 (1.74)	0.461 ± 0.042 (14.10)^***^	0.003 ± 0.001 (11.33)^***^	0.286 ± 0.028 (316.78)^***^
Docetaxel		0.206 ± 0.044 (1.00)	22.546 ± 1.581 (1.00)	0.010 ± 0.001 (1.00)	75.711 ± 9.275 (1.00)
	+10 μM TTM	0.198 ± 0.038 (1.04)	5.956 ± 1.692 (3.79)^*^	0.007 ± 0.001 (1.43)	1.969 ± 0.010 (38.45)^***^
	+30 μM TTM	0.163 ± 0.048 (1.26)	1.250 ± 0.497 (18.04)^***^	0.005 ± 0.001 (1.40)	1.163 ± 0.030 (65.10)^***^
	+10 μM Ver	0.146 ± 0.012 (1.41)	2.403 ± 0.980 (9.38)^**^	0.005 ± 0.001 (1.40)	0.087 ± 0.031 (870.24)^***^
Cisplatin		6.763 ± 0.56 (1.00)	3.605 ± 0.494 (1.00)	28.478 ± 4.580 (1.00)	32.956 ± 1.455 (1.00)
	+10 μM TTM	5.806 ± 0.59 (1.16)	3.521 ± 0.361 (1.02)	23.122 ± 2.812 (1.23)	29.732 ± 3.591 (1.11)
	+30 μM TTM	5.474 ± 0.57 (1.24)	3.281 ± 0.389 (1.10)	19.492 ± 3.181 (1.46)	24.488 ± 4.600 (1.35)
	+10 μM Ver	5.379 ± 0.74 (1.25)	3.222 ± 0.346 (1.12)	15.217 ± 1.175 (1.87)	20.964 ± 3.308 (1.572)

Cells were treated with a various concentrations of conventional chemotherapeutic drugs in combination with or without TTM and analyzed by MTT and CCK-8 assays.

^a^ Data are presented as mean ± SEM(n=3) of at least three independent experiments.

^b^ Reversal fold change (RF) = IC_50_ (MDR cells) / IC_50_ (MDR cells treated with TTM or Ver treatment). ^*^ denotes *P* < 0.05, ^**^ denotes *P* < 0.01 and ^***^ denotes *P* < 0.001 compared to control or untreated group IC_50_.

Since overexpression of ABC transporters is the primary determinant of the MDR phenotype, we examined the expression of three main ABC transporters, *MDR1, MRP1 and BCRP* by qRT-PCR. As shown in Figure 1B, *MDR1* mRNA was highly expressed in MCF-7/MDR and K562/MDR cells than the corresponding parental cells. However, MRP1 or BCRP mRNA levels were similar in both MDR and parental MCF-7 and K562 cells (Figure 1B).

The chemotherapy agents including DOX, DNR, EPI and Doc are exported out of the cells by P-gp [32–34]. Therefore, we examined the levels of P-gp protein in MDR and parental MCF-7 and K562 cells. Our data showed high P-gp protein levels in MCF-7/MDR and K562/MDR cells than the corresponding parental cells (Figure 1C) and was consistent with the previous study [35, 36]. Therefore, we selected MCF-7/MDR and K562/MDR cells to investigate if TTM reversed P-gp-mediated MDR.

### TTM is not cytotoxic to MDR cancer cells and non-tumor cells

We determined cytotoxicity of TTM on MDR and parental MCF and K562 cells by MTT and Cell Counting Kit-8 (CCK-8) assays. We observed that TTM concentrations below 100 μM did not significantly inhibit proliferation of MDR and parental MCF and K562 cells (Figure 1D and 1E). Moreover, treatment with 0-100 μM TTM did not affect cell survival in MDR and parental MCF-7 and K562 cells between 0-48 hrr (Figure 1D and 1E). We also showed that 0-100 μM TTM showed no cytotoxicity on non-cancer human mammary epithelial MCF-10A cells at 48 hr (Figure 1F). Therefore, we selected 10 μM and 30 μM TTM for further experiments since they showed minimal growth inhibition of <5% and <10%, respectively.

### TTM reverses P-gp-mediated MDR *in vitro*

We investigated if TTM treatment enhanced chemosensitivity of MDR cells to anticancer drugs such as DOX, DNR, EPI, Doc and DDP. We used Verapamil, a P-gp inhibitor and calcium channel blocker as a positive control [37–39]. TTM decreased the drug resistance in MDR cells in a dose-dependent manner. As shown in Table 1, IC_50_ for DOX, DNR, EPI and Doc decreased by 7.26-, 2.81-, 3.68-, 18.04-fold for MCF-7/MDR and 5.76-, 32.68-, 47.21-, 65.10-fold for K562/MDR cells, when treated with 30 μM TTM. On the other hand, IC_50_ for DOX, DNR, EPI and Doc decreased by 2.39-, 1.42-, 1.77-, 3.79-fold for MCF-7/MDR and 3.38-, 3.04-, 11.97- , 38.45-fold for K562/MDR, when treated with 10 μM TTM. Moreover, TTM treatment did not change the IC_50_ values of DOX, DNR, EPI, Doc and DDP in the parental MCF-7 and K562 cells (Table 1). These results indicate that TTM reverses P-gp mediated MDR for DOX, DNR, EPI and Doc.

### TTM increases apoptosis in Doc-treated MDR cancer cells

**Figure 2:**
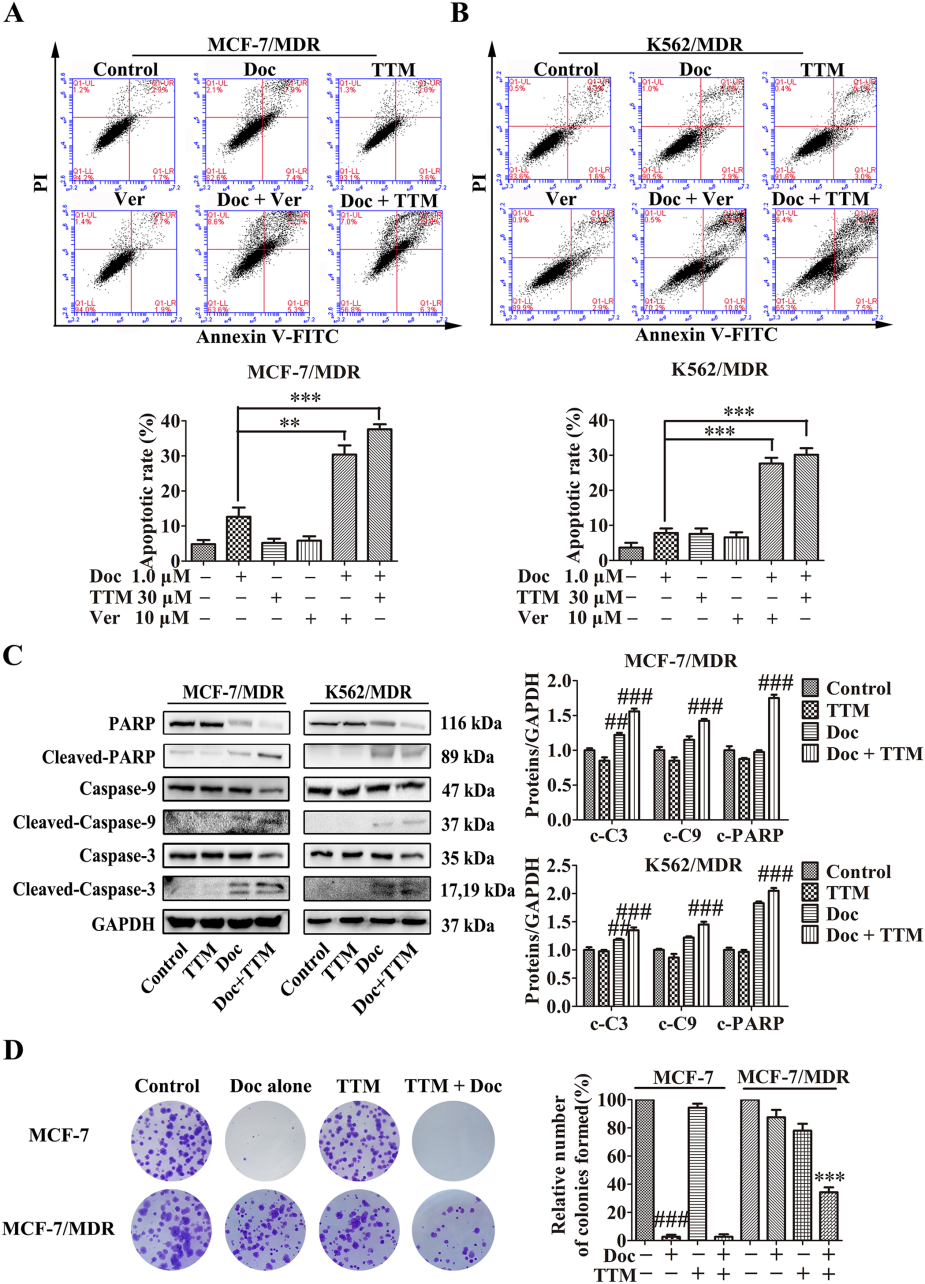
Effect of TTM on apoptosis and clonogenicity of Doc-treated MDR cancer cells **(A-B)** Flow cytometry analysis of cellular apoptosis in (A) MCF-7/MDR and (B) K562/MDR cells treated with 1 μM Doc, 30 μM TTM, 10 μM Ver, or combination of 1 μM Doc plus 10 μM Ver plus 30 μM TTM for 48 hr as determined by AnnexinV-FITC/PI staining. Note: Data represent three independent experiments. As shown, MDR cells were resistant to Doc, and both TTM and Ver enhanced Doc-induced apoptosis. ^**^ denotes *P* < 0.01 and ^***^ denotes *P* < 0.001 compared to Doc alone treatment. **(C)** Quantitative western blot analysis of procaspase-9, cleaved caspase-9, procaspase-3, cleaved caspase-3, PARP and cleaved PARP in MCF-7/MDR and K562/MDR cells incubated with 1 μM Doc with or without 30 μM TTM for 48 hr. Note: GAPDH was used as internal control. ^##^ denotes *P* < 0.01 and ^###^ denotes *P* < 0.001 compared to control. **(D)** Histogram plots showing total number of colonies in MCF-7 and MCF-7/MDR cells treated with 1 μM Doc with or without 30 μM TTM for 48 hr. Note: Cells were plated in 6-well plates containing RPMI 1640 plus 10% FBS at a density of 1000 cells per well. Colonies were counted after 15 days. The data are presented as the mean ± SEM from three independent experiments. ^###^ denotes *P* < 0.001 compared to control. ^***^ denotes *P* < 0.001 compared to Doc alone treatment.

We evaluated the effects of TTM on Doc-induced apoptosis. As shown in Figure 2A-2B, MDR cells treated with (+) or without (-) 30 μM TTM or 10 μM Ver combined with 1 μM Doc for 48 hr. The results indicated that treatment of the MDR cells with 1 μM Doc, especially with 30 μM TTM or 10 μM Ver alone, did not cause notable apoptosis, while combination treatment dramatically increased Doc-induced apoptosis (30.4% ± 3.68% and 37.6% ± 1.97% for Ver and TTM combined with Doc respectively, versus 12.65% ± 3.74% for Doc alone in MCF-7/MDR cells; 27.65% ± 2.33% and 30.15% ± 2.62% for Ver and TTM combined with Doc respectively, versus 7.8% ± 1.84% for Doc alone in K562/MDR cells). In the parental MCF-7 and K562 cells, Doc treatment alone induced significant cell death (Supplementary Figures 1 and 2). Moreover, TTM increased Doc-induced apoptosis in K562 parental cells, but had no effect on MCF-7 cells.

Caspases are important mediators of cellular apoptosis that cleave various proteins that are necessary for cell survival and function [40]. We assessed the status of cleaved caspases 3 and 9 as well as PARP in MDR cells treated with TTM and Doc in combination. As shown in Figure 2C, MCF-7/MDR and K562/MDR cells treated with a combination of Doc and TTM showed increased cleaved caspases-3, -9 and PARP levels. This suggested that TTM treatment enhances Doc-induced apoptosis in MDR cells by activating the apoptotic machinery.

### TTM decreases clonogenicity of Doc or DOX-treated MDR cancer cells

MDR cancer cells with high P-gp expression are highly malignant and resistant to traditional chemotherapeutic agents [41, 42]. Therefore, we tested the effects of TTM on clonogenicity of parental and MDR MCF-7 cells treated with 1 μM Doc or DOX with or without 30 μM TTM. MCF-7/MDR cells formed greater number of colonies than the parental MCF-7 cells (Figure 2D and Supplementary Figure 3). However, combined treatment of MCF-7/MDR cells with 1 μM Doc or 1 μM DOX in presence of 30 μM TTM decreased the colonies by 53.2% and 68.1%, respectively (Figure 2D Column 4 and Supplementary Figure 3 Column 4). However, parental MCF-7 cells treated with 1 μM Doc or 1 μM DOX or 1 μM Doc/1 μM DOX with 30 μM TTM showed no difference in colony numbers (Figure 2D Column 2 and Supplementary Figure 3 Column 2). These results demonstrated that TTM enhanced the cytotoxicity of Doc or DOX treated MDR cancer cells by decreasing their colony formation ability.

### TTM enhances the accumulation of DOX and Rh123 in MDR cells

**Figure 3:**
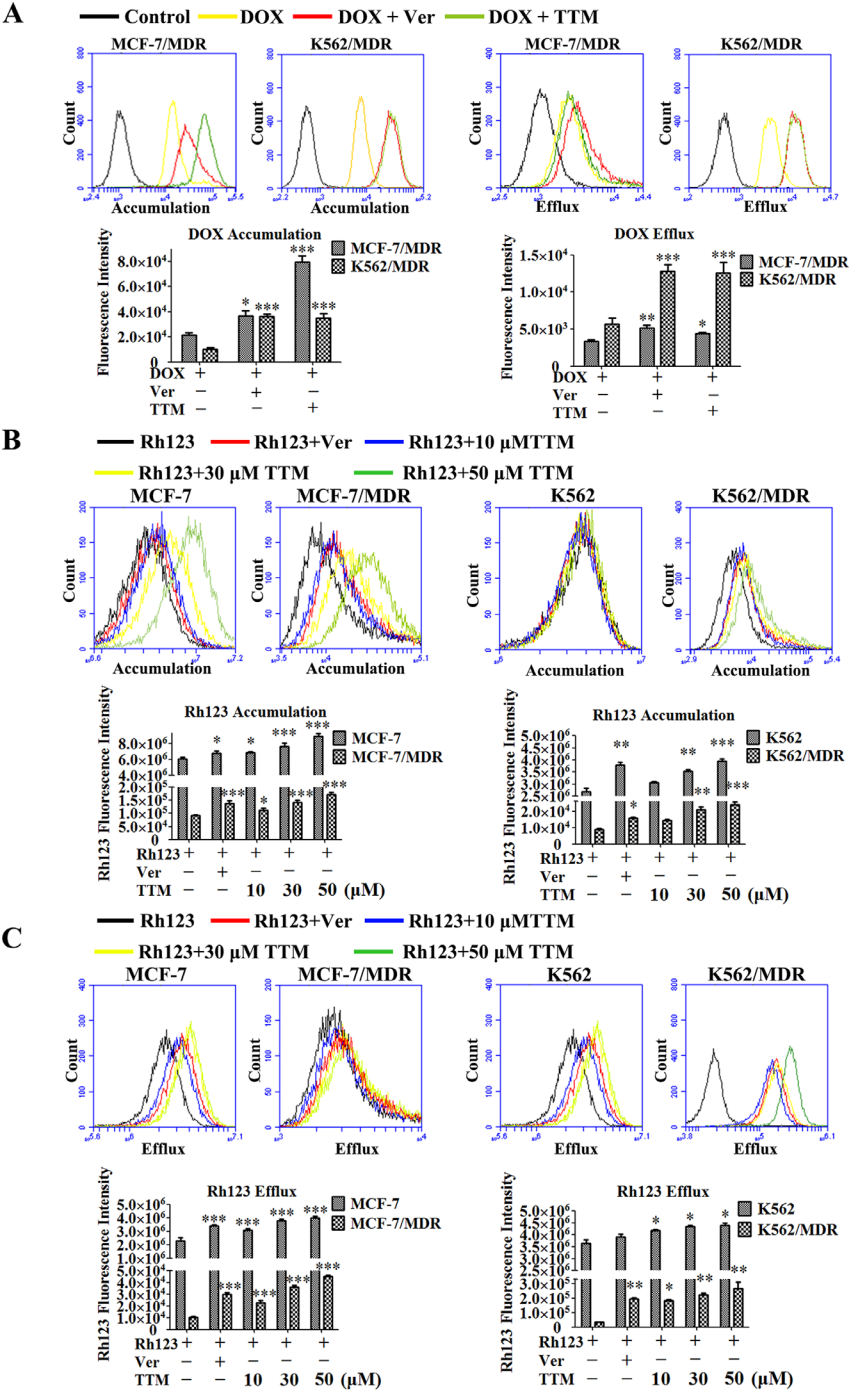
Effect of TTM on the accumulation and efflux of DOX and Rh123 in MDR cancer cells **(A)** Histogram plots showing flow cytometry analysis of intracellular DOX accumulation in MCF-7/MDR and K562/MDR cells pre-incubated for 4 hr with or without 10 μM TTM or 10 μM Ver (positive control) followed by incubation with 10 μM DOX as determined by flow cytometry. Also shown are histogram plots for DOX retention assay, wherein MDR cells were pre-incubated for 3 hr with 10 μM DOX followed by incubation for 4 hr in medium with or without 10 μM TTM or 10 μM Ver. Subsequently, intracellular DOX levels were determined by flow cytometry. **(B)** Histogram plots showing flow cytometry analysis of Rh123 dye accumulation in MDR and parental MCF-7 and K562 cells pretreated with or without 10, 30, 50 μM TTM or 10 μM Ver for 1.5 hr followed by incubation with 5 μM Rh123 in the dark for 1.5 hr. **(C)** Histogram plots showing flow cytometry analysis of Rh123 efflux in MDR and parental MCF-7 and K562 cells that were first incubated with 5 μM Rh123 in the dark for 1.5 hr followed by incubation with or without 10, 30, 50 μM TTM or 10 μM Ver for 1.5 hr. Intracellular fluorescence of Rh123 was detected by flow cytometry. Note: The data are represented as the mean ± SEM from three independent experiments. ^*^ denotes *P* < 0.05, ^**^ denotes *P* < 0.01 and ^***^ denotes *P* < 0.001 compared to DOX or Rh123 alone treatments.

We next determined the effects of TTM on drug accumulation in MDR cancer cells by fluorescent detection of DOX and Rh123 dye in the presence and absence of TTM and Ver. Treatment with 10 μM TTM and 10 μM Ver increased intracellular levels of DOX by 3.71- and 1.71-fold in MCF-7/MDR cells, and 3.48- and 3.61-fold in K562/MDR cells, respectively (Figure 3A). The efflux of DOX was reduced by 1.32 and 1.54-fold in MCF-7/MDR cells, and 2.22 and 2.27-fold in K562/MDR cells upon treatment with 10 μM TTM and 10 μM Ver, respectively (Figure 3A). Moreover, treatment with 10 μM TTM and 10 μM Ver increased DOX accumulation by 1.33- and 1.16-fold in MCF-7 cells and 1.31- and 1.26-fold in K562 cells, respectively (Supplementary Figure 4). Treatment with 10 μM TTM and 10 μM Ver reduced DOX efflux by 1.47- and 2.30- fold in MCF-7 cells, and 1.26- and 1.17-fold in K562 cells, respectively (Supplementary Figure 4).

Since P-gp plays an important role in DOX efflux, we examined the effects of TTM by assessing the accumulation and efflux of Rh123, which is a specific P-gp substrate [43]. We found that treatment with 10 μM Ver and 10, 30 or 50 μM TTM increased Rh123 accumulation by 1.49-, 1.22- , 1.53- and 1.87-fold in MCF-7/MDR cells, 1.11-, 1.12-, 1.25- and 1.46-fold in MCF-7 cells, 1.28-, 1.20-, 1.31- and 1.39-fold in K562/MDR and 1.20-, 1.06-, 1.16- and 1.27-fold in K562 cells, respectively (Figure 3B). Moreover, as shown in Figure 3C, treatment with 10 μM Ver and 10, 30 or 50 μM TTM reduced Rh123 efflux by 2.84-,2.15-,3.42- and 4.26-fold in MCF-7/MDR, 1.50-, 1.36-,1.67- and 1.76-fold in MCF-7, 5.85-, 5.45-, 6.72- and 7.94-fold in K562/MDR, 1.07-,1.14-, 1.19- and 1.20-fold in K562 cells, respectively. Furthermore, laser confocal microscopy showed that TTM enhanced cytosolic accumulation of Rh123 in MCF-7/MDR and K562/MDR cells than controls (Supplementary Figure 5). These results demonstrated that TTM increased intracellular concentrations of DOX by inhibiting P-gp efflux function in MDR cells.

### TTM decreases P-gp levels in MDR cells

**Figure 4:**
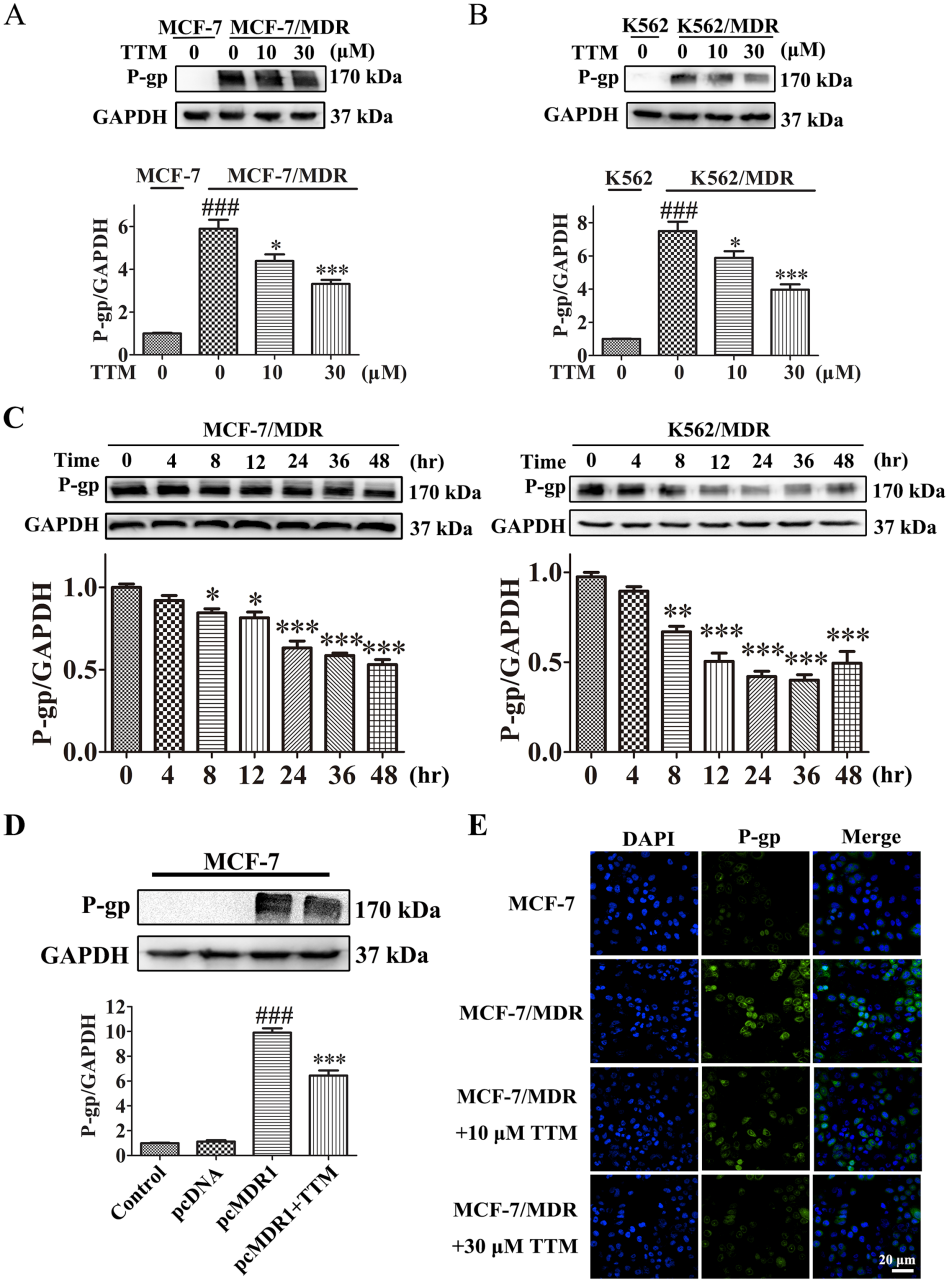
TTM decreases P-gp levels in MDR cancer cells **(A)** Representative western blot showing P-gp expression in MCF-7/MDR cells treated with 0 to 30 μM TTM for 48 hr. MCF-7 cells were used as the negative control. ^###^ denotes *P* < 0.001 compared to MCF-7. ^*^ denotes *P* < 0.05 and ^***^ denotes *P* < 0.001 compared to MCF-7/MDR. **(B)** Representative western blot showing P-gp expression in K562/MDR cells treated with 0 to 30 μM TTM for 48 hr. K562 cells were used as the negative control. ^###^ denotes *P* < 0.001 compared to K562. ^*^ denotes *P* < 0.05 and ^***^ denotes *P* < 0.001 compared to K562/MDR. **(C)** Representative western blot analysis of P-gp levels in MCF-7/MDR and K562/MDR cells treated with 30 μM TTM at 0, 4, 8, 12, 24, 36 and 48 hr. ^*^ denotes *P* < 0.05, ^**^ denotes *P* < 0.01 and ^***^ denotes *P* < 0.001 are all in comparison to control. **(D)** Representative western blot analysis of P-gp protein levels in MCF-7 cells transfected with control or P-gp overexpression plasmid for 24 hr followed by treatment with or without 30 μM TTM for 48 hr. Note: ^###^ denotes *P* < 0.001 versus pcDNA and ^***^ denotes *P* < 0.001 versus pcMDR1. **(E)** Representative confocal microscopy images showing subcellular localization of the P-gp protein in MCF-7/MDR and MCF-7 cells treated with or without 10 or 30 μM TTM for 48 hr. The P-gp immunostaining is shown in green and the DAPI stained nuclei are shown in blue. Scale bar equals 20 μM. Quantitative data are presented as the mean ± SEM from three independent experiments.

Next, we analyzed if TTM decreased P-gp expression in MDR cells. The P-gp protein levels decreased in a dose-dependent manner in MCF-7/MDR and K562/MDR cells when treated with 0-30 μM TTM for 48 hr (Figure 4A-4B). Moreover, P-gp levels decreased in a time-dependent manner when treated 30 μM TTM for 48 hr (Figure 4C). We transfected MCF-7 cells with P-gp overexpression plasmid to determine if TTM effects on MDR were related to P-gp protein levels. We observed that 30 μM TTM decreased P-gp levels in MDR1-overexpressing MCF-7 cells (Figure 4D). We also showed by laser scanning confocal microscopy that P-gp levels were higher in MCF-7/MDR cells than MCF-7 cells; TTM reduced P-gp levels in MCF-7/MDR cells in a concentration dependent manner (Figure 4E). These data demonstrated that TTM decreased MDR by lowering P-gp expression.

### TTM inhibits p38 MAPK signaling

**Figure 5:**
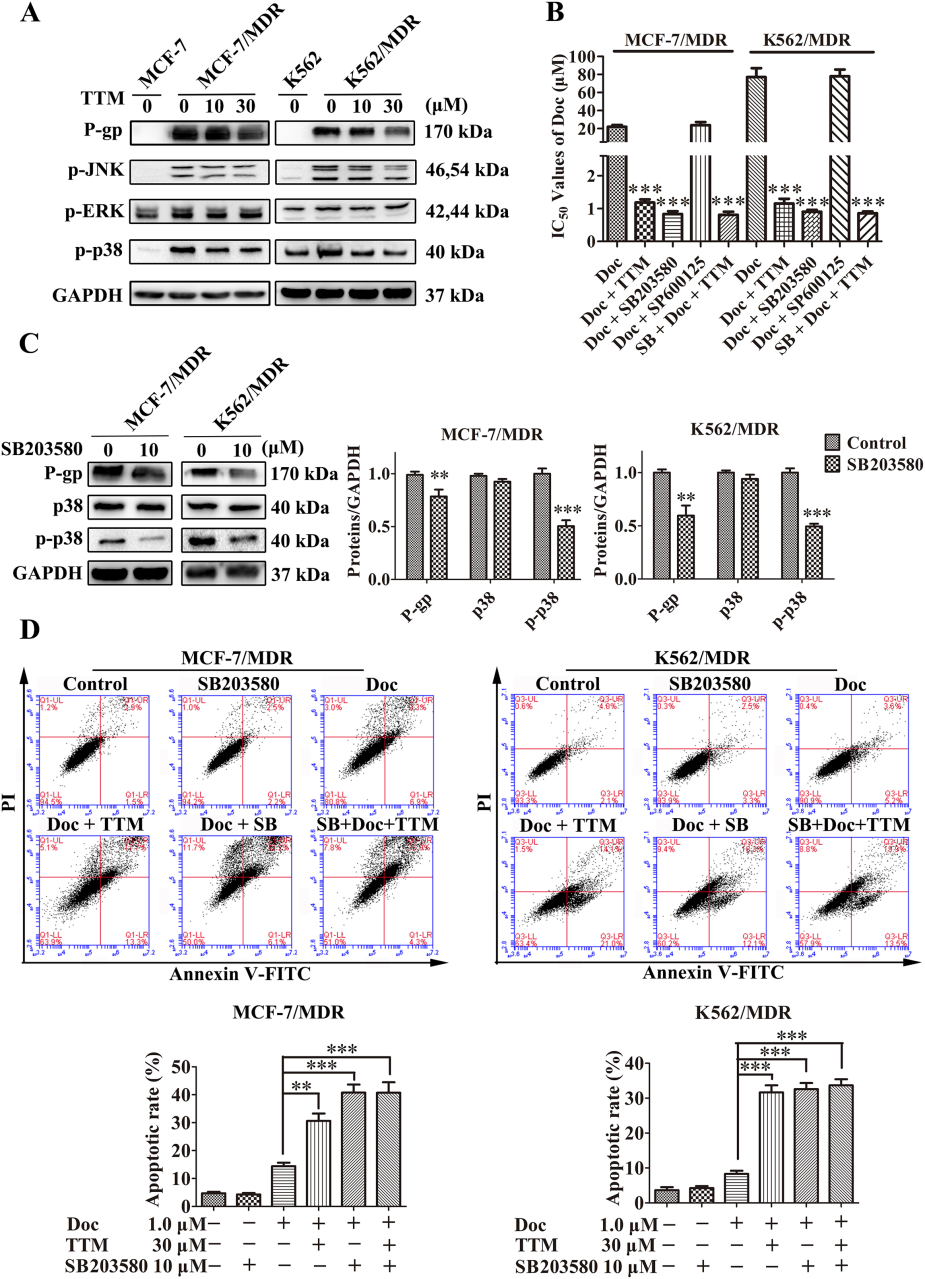
TTM reduces P-gp expression by suppressing p38 MAPK activation in MDR cancer cells **(A)** Representative western blot analysis of P-gp and total and phosphorylated ERK, JNK and p38 MAPK proteins in MDR and parental MCF-7 and K562 cells treated with 10 or 30 μM TTM for 48 hr. GAPDH was used as a loading control. **(B)** MTT assay results showing cell viability of MCF-7/MDR or K562/MDR cells treated with the different concentrations of Doc in the absence or presence of 10 μM SB203580, 10 μM SP600125 and/or 30 μM TTM for 48 hr. ^***^ denotes *P* < 0.001 compared to Doc alone treated controls. **(C)** Representative western blot analysis of P-gp, p38 MAPK and phospho-p38 MAPK expression in MCF-7/MDR and K562/MDR cells treated with 10 μM SB203580 for 48 hr. ^**^ denotes *P* < 0.01 and ^***^ denotes *P* < 0.001 compared to control. **(D)** Flow cytometry analysis of cell death by AnnexinV-FITC/PI staining of MCF-7/MDR and K562/MDR cells incubated with 1 μM Doc and/or 30 μM TTM and/or 10 μM SB203580. The data are represented as percentage of apoptotic cells (AnnexinV^+^ PI^+^ and AnnexinV^+^ PI^-^). Note: ^**^ denotes *P* < 0.01 and ^***^ denotes *P* < 0.001 compared to the Doc alone treated cells. All experiments were repeated three times and data are represented in the histogram as mean ± SEM.

P-gp expression is regulated by MAPK signaling pathway and therefore inhibition of MAPK signaling is an effective therapeutic approach to overcome MDR [44]. Therefore, we analyzed the phosphorylation levels of JNK, ERK and p38 MAPKs in TTM-treated MDR cells. First, we observed increased phospho-p38 and phospho-JNK levels in MCF-7/MDR and K562/MDR cells than the parental MCF-7 and K562 cells (Figure 5A). Moreover, TTM treatment decreased phospho-p38 and phospho-JNK levels, whereas phospho-ERK levels remained unchanged in MDR cells (Figure 5A).

Next, we used the MTT assay to determine the effects of JNK inhibitor SP600125 and p38 MAPK inhibitor SB203580 on Doc-treated MCF-7/MDR cells. We observed that p38 MAPK inhibitor, SB203580 decreased IC_50_ for Doc in MCF-7/MDR cells, whereas JNK inhibitor, SP600125 had no effect (SB203580: 0.83 ± 0.09 μM; SP600125: 23.76 ± 3.43 μM; no treatment: 21.88 ± 2.00 μM; Figure 5B). We further observed that TTM treatment did not further decrease IC_50_ of Doc in presence of SB203580 (SB203580 plus TTM: 0.81 ± 0.09 μM; Figure 5B). In K562/MDR cells, similar results were obtained for Doc cytotoxicity (SB203580: 0.9 ± 0.1 μM; SP600125: 77.97 ± 7.62 μM; Doc only: 77.23 ± 9.82 μM; SB203580 plus TTM: 0.85 ± 0.10 μM; Figure 5B). This suggested that TTM decreased P-gp levels by inhibiting p38 MAPK. SB203580 decreased Doc resistance of MDR cells more effectively than TTM and did not inhibit cell proliferation at the concentrations used in our assays (Supplementary Figure 6).

Next, we tested the effects of 10 μM SB203580 in MDR cells. SB203580 reduced phospho-p38 MAPK levels in both MCF-7/MDR and K562/MDR cells (Figure 5C). We observed 1.38- and 1.67-fold reduced P-gp expression in SB203580 treated MCF-7/MDR and K562/MDR cells than the corresponding untreated cells (Figure 5C). This demonstrated that the p38 MAPK pathway regulated P-gp expression.

We then investigated if SB203580 treatment enhanced apoptosis in Doc treated MCF-7/MDR and K562/MDR cells by FACS analysis of AnnexinV-FITC/PI staining. SB203580 treatment alone did not induce apoptosis, but, it reduced phospho-p38 levels in MDR cells (Figure 5D and Figure 5C). We observed slight karyopyknosis by laser confocal microscopy in MCF-7 and MCF-7/MDR cells treated with 10 μM SB203580 alone (data not shown). However, when MDR cells were pretreated with 10 μM SB203580 for 1 h, they demonstrated increased apoptosis when treated with Doc than Doc alone (40.77% ± 2.87% for SB203580 combined with Doc, versus 14.4% ± 1.25% for Doc alone in MCF-7/MDR cells; 32.6% ± 1.75% for SB203580 combined with Doc, versus 8.33% ± 0.88% for Doc alone in K562/MDR cells) (Figure 5D). TTM did not further enhance apoptosis induced by SB203580 in Doc-treated MDR cells (Figure 5D). These results indicated that inhibition of p38 MAPK enhanced chemosensitivity of MDR cells by activating apoptosis.

### TTM inhibits p38 MAPK-induced P-gp in MCF-7 and K562 cells

**Figure 6:**
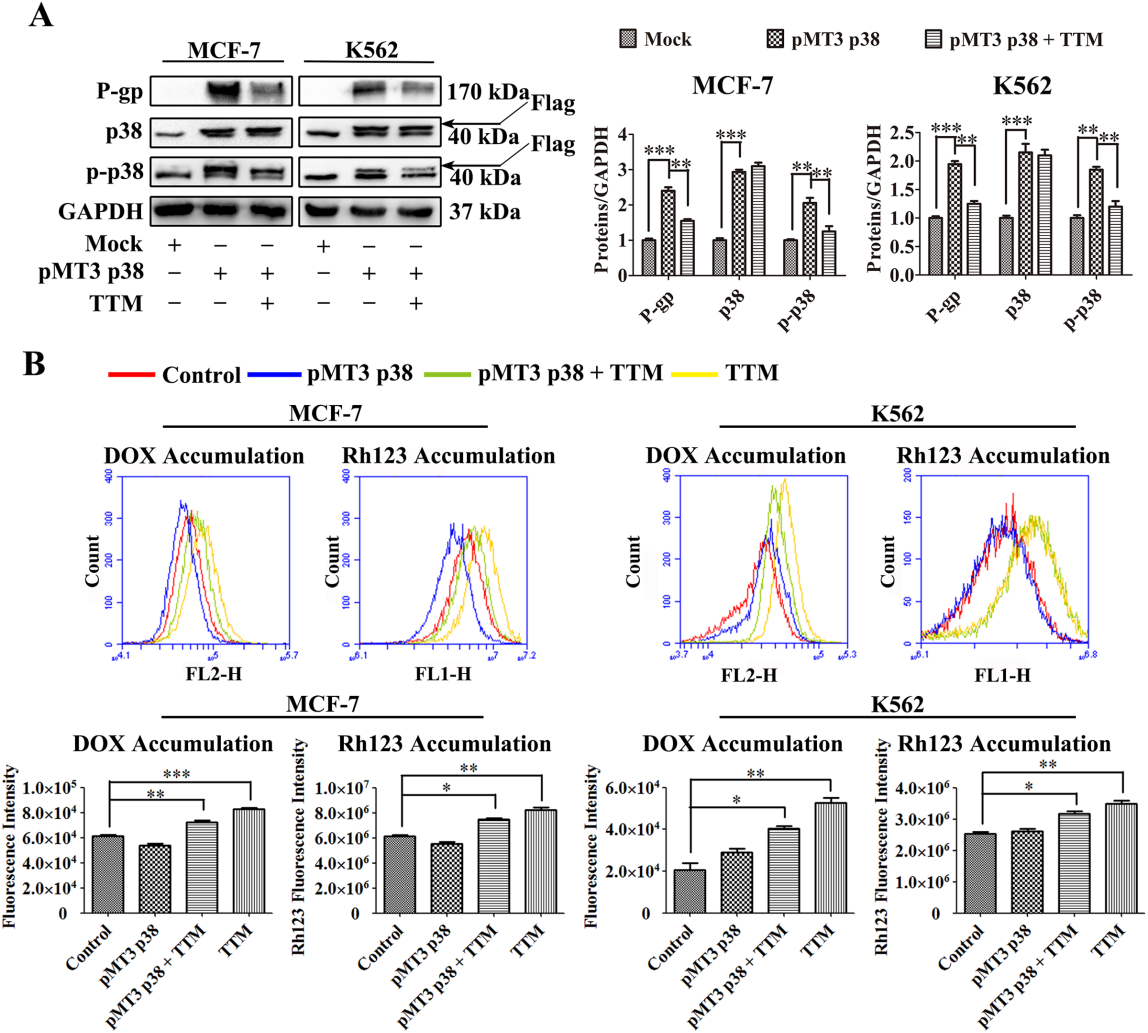
Effect of TTM on p38 MAPK-mediated increase in P-gp expression in parental MCF-7 and K562 cells **(A)** Representative western blot analysis of P-gp and total and phosphorylated p38 MAPK levels in control and p38 MAPK overexpressing MCF-7 and K562 cells treated with or without 30 μM TTM for 48 hr. MCF-7 and K562 cells were transfected with (+) or without (-) 10 μg/l pMT3-p38MAPK plasmid for 24 hr to increase p38 MAPK expression. GAPDH was used as a loading control. Note: ^**^ denotes *P* < 0.01 and ^***^ denotes *P* < 0.001 compared to the pMT3 p38 group. **(B)** Flow cytometry analysis of DOX and Rh123 accumulation in control and p38 MAPK overexpressing MCF-7 and K562 cells treated with or without 30 μM TTM for 48 hr. After 48 hr, the cells were incubated with 10 μM DOX for 4 hr or 5 μM Rh123 for 2 hr. Intracellular fluorescence was analyzed by flow cytometry. The data are presented as the mean ± SEM from three independent experiments. Note: ^*^ denotes *P* < 0.05, ^**^ denotes *P* < 0.01 and ^***^ denotes *P* < 0.001 compared to the control group.

Finally, we verified if TTM decreased P-gp expression in MDR cells by inhibiting p38 MAPK signaling pathway. First, we transfected MCF-7 and K562 parental cells with p38 overexpression vector (pMT3 p38) for 24 hr. Then, we treated the cells with or without TTM for an additional 48 hr and examined the levels of P-gp, p38 and phospho-p38. Western blot analysis demonstrated that total and phosphorylated p38 MAPK as well as P-gp levels were higher in p38 MAPK-overexpressing MCF-7 and K562 cells that were not treated with TTM (Figure 6A). However, TTM-treated p38 MAPK-overexpressing MCF-7 and K562 cells showed decreased levels of phospho-p38 and P-gp (Figure 6A). Moreover, qRT-PCR data demonstrated that TTM inhibited *MDR1* mRNA levels in p38 MAPK-overexpressing MCF-7 and K562 cells (Supplementary Figure 7). We also observed that p38 MAPK-overexpressing MCF-7 and K562 cells treated with TTM showed increased DOX and Rh123 accumulation (Figure 6B). These results confirmed that TTM decreased P-gp levels by inhibiting p38 MAPK signaling.

## DISCUSSION

In this study, we investigated the mechanism of action by which TTM, a novel meroterpenoid that is isolated from the leaves of *Rhodomyrtus tomentosa* reverses P-gp-mediated MDR in cancer cells. This is the first report of a syncarpic acid-conjugated terpenoid as a MDR modulator.

We demonstrated that TTM decreased the high expression of P-gp in MCF-7/MDR and K562/MDR cells, thereby reversing multidrug resistance within acceptable levels of cytotoxicity [27]. We demonstrated that TTM below 100 μM did not inhibit cell proliferation in tumor and non-tumor cells (Figure 1D-1F), but, enhanced cytotoxicity of different chemotherapeutic drugs, DOX, DNR, EPI and Doc in the P-gp overexpressing cancer cell lines.

The ABC transporter proteins that mediate MDR are involved in cellular detoxification [20]. They pump out cytotoxic drugs from cancer cells, thereby decreasing their intracellular levels and thus enhance tumor cell survival. Therefore, P-gp antagonists re-sensitize MDR cells to anti-cancer drugs [45]. We demonstrated that TTM increased docetaxel cytotoxicity in MDR cancer cells by enhancing apoptosis as shown by high cleaved-caspase-9/3 and cleaved-PARP levels (Figure 2A-2C). TTM treatment alone did not affect cell viability.

Increased clonogenicity is characteristic of malignant and drug-resistant cancer cells, which are responsible for cancer recurrence [46]. We demonstrated that MDR cells were more clonogenic and resistant to anticancer drugs, Doc or DOX. However, TTM in combination with Doc or DOX significantly reduced clonogenicity of MDR cancer cells (Figure 2D and Supplementary Figure 3). We further demonstrated that TTM enhanced intracellular accumulation of DOX and Rh123 in MDR cells by reducing their efflux in a concentration-dependent manner (Figure 3, Supplementary Figure 4 and Supplementary Figure 5).

Natural products inhibit MDR by either preventing efflux activity of P-gp or downregulating P-gp/MDR1 expression [47]. We observed that TTM significantly reduced P-gp expression in MCF-7/MDR and K562/MDR cells. Since P-gp has a long half-life [23], we examined the time-course of TTM downregulation of P-gp in the two MDR cancer cells (Figure 4C). We also found that TTM blocked *MDR1* mRNA levels in the two MDR cancer cells (data not shown). This is the first report demonstrating the ability of TTM, a natural syncarpic acid-conjugated monoterpene in reducing the levels of MDR transporter, P-gp. We observed that decreased P-gp levels were observed after 8 hr of TTM treatment in MDR cells, although increased Rh123 accumulation was observed at 4 hr post-TTM treatment. This suggests that TTM inhibits P-gp function.

Anticancer drugs activate or inhibit signaling pathways that are associated with drug resistance of tumor cells [48]. ABC transporter expression has been associated with MAPK signaling [49, 50]. The MAPK signaling pathway is an important player in MDR and a promising target for systemic therapy [5, 51]. Moreover, the p38 signaling pathway is associated with cellular apoptosis in cancer cells [39, 52]. We demonstrated that MDR cells showed high phospho-p38 levels that correlated with P-gp overexpression in MCF-7/MDR and K562/MDR cells than the corresponding parental cells (Figure 5A). We further demonstrated that TTM decreased phopho-p38 MAPK and P-gp levels in a concentration-dependent manner. Moreover, the p38 MAPK inhibitor, SB203580 decreased Doc resistance of the MDR cancer cells. TTM did not alter the cytotoxicity of Doc upon SB203580 pre-treatment of MDR cancer cells (Figure 5B and Supplementary Figure 6). Moreover, SB203580 pre-treatment decreased P-gp levels in MDR cells. However, SP600125, a JNK inhibitor had no effect in combination with Doc or DOX (Figure 5B). Hence, we showed that p38 MAPK promotes P-gp-mediated MDR. SB203580 also enhanced Doc-induced apoptosis in MDR cancer cells (Figure 5D). These results demonstrate that the p38 MAPK pathway enhances P-gp levels in MCF-7/MDR and K562/MDR cells.

P-gp stability is correlated with the activator protein-1 (AP-1) transcription factor [52], which is targeted by MAPK signaling pathways [53]. Human MDR1 promoter contains an AP-1-binding site [54], and increased AP-1 binding has been observed in several multidrug-resistant cell lines [55], whereas, reduced AP-1 binding has been associated with increased drug sensitivity in other cancer cell lines [56, 57]. SB203580 has been shown to block c-Fos and c-Jun expression in response to UV irradiation and anisomycin [58]. Although p38 MAPK does not directly phosphorylate or activate c-Jun, several lines of evidence support the idea that p38 MAPK contributes to c-Jun induction via AP-1, which binds in the c-Jun promoter [59, 60]. In addition to AP-1, c-Jun gene expression is regulated by MEF2 family of transcription factors [61]. MEF2 is essential for LPS induction of c-Jun in macrophages, which also requires functional p38 MAPK and MEF2C transcriptional factor. Furthermore, MEF2C is directly phosphorylated and activated by p38 MAPK [62]. Thus, p38 MAPK can potentially modulate c-Jun transcriptional activity by regulating AP1 and MEF2 transcription factors that have binding sites in *c-Jun*. We postulate that cJun may be intrinsically involved in p38 MAPK mediated P-gp expression.

It is also worth noting that SB203580 showed more cytotoxicity than TTM due to induction of karyopyknosis induction (data not shown) and lower IC_50_ values in our models (Figure 1D–1F, and Supplementary Figure 6). We further demonstrated that p38 overexpression parental MCF-7 and K562 cells increased P-gp levels by enhancing phospho-p38 MAPK levels. Thus, higher p38 MAPK levels decreased efficacy of TTM-mediated P-gp downregulation (Figure 6A and Supplementary Figure 7). Moreover, higher p38 MAPK levels decreased intracellular DOX and Rh123 levels in presence of TTM (Figure 6B).

**Figure 7:**
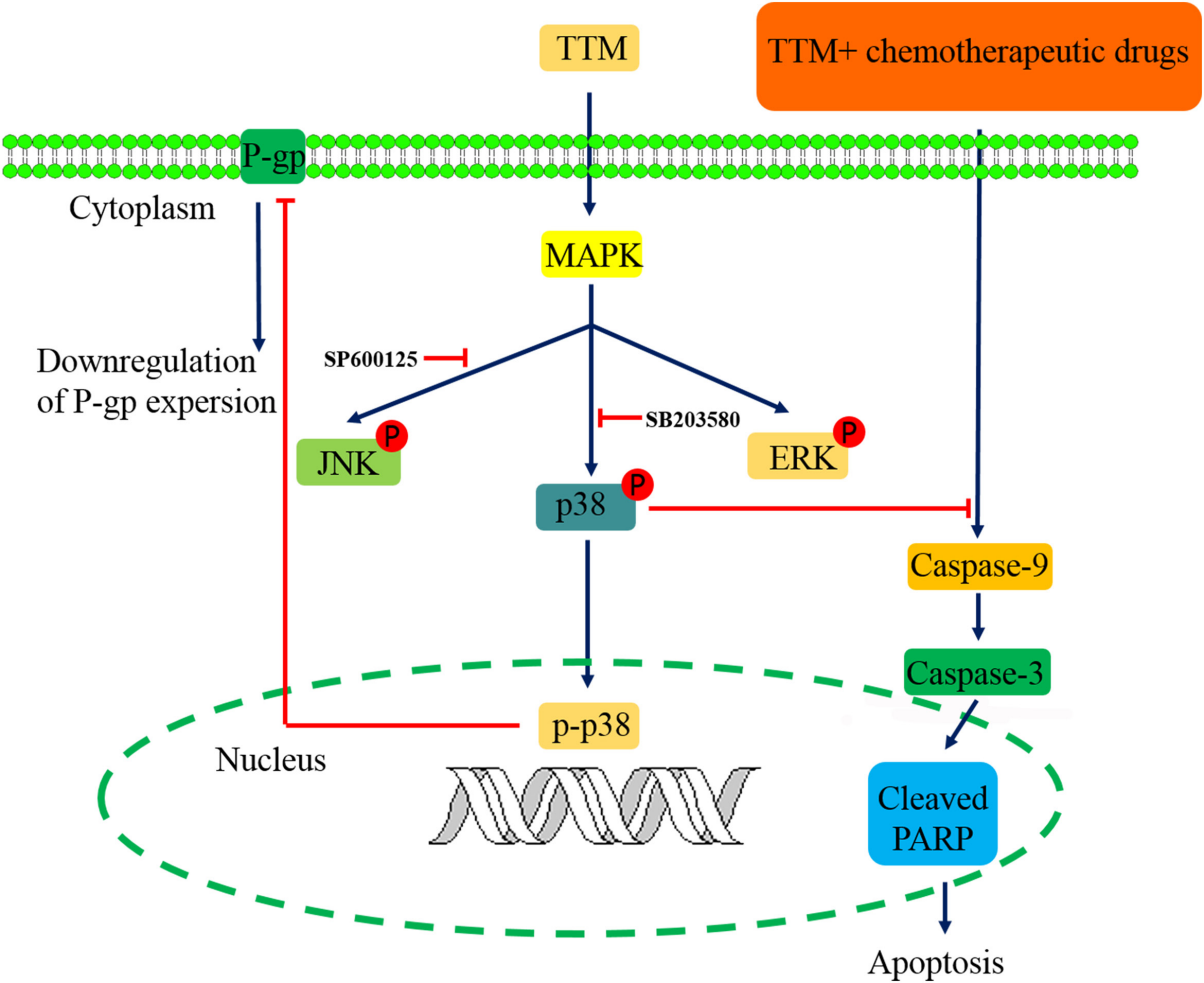
Schematic diagram of TTM-modulated signaling pathways that reverse multidrug resistance in MCF-7/MDR and K562/MDR cells

In conclusion, TTM, a novel syncarpic acid-conjugated monoterpene, increases cytotoxicity of chemotherapeutic drugs in MDR cancer cells by decreasing P-gp expression via p38 MAPK inhibition (Figure 7). Thus, our study demonstrates that TTM is a potential therapeutic drug for cancer patients demonstrating multi-drug resistance.

## MATERIALS AND METHODS

### Isolation and identification of TTM from *Rhodomyrtus tomentosa*

TTM was isolated from the petroleum ether soluble fraction of 95% ethanol extract of the leaves of *Rhodomyrtus tomentosa* through various chromatographic techniques including column chromatography over silica gel, ODS, Sephadex LH-20 and preparative HPLC. The structure was elucidated as a novel syncarpic acid-conjugated monoterpenoid by a comprehensive analysis of the spectral data (IR, UV, HRESIMS, ECD, 1D- and 2D-NMR). The detailed isolation and structural elucidation are shown in the latest article [63].

### Materials

DOX was purchased from Santa Cruz Biotechnology (Santa Cruz, CA). DNR and EPI were purchased from National Institutes for Food and Drug Control (Beijing, China). Doc was from ApexBio (Houston, USA). DDP, Ver, MTT, Rh123, paraformaldehyde, bovine serum albumin (BSA), Tris, dimethylsulfoxide (DMSO), NaCl, EDTA, PMSF, SDS, and DTT were purchased from Sigma-Aldrich (St. Louis, MO). Opti-MEM and fetal bovine serum (FBS) were purchased from Life Technologies (Grand Island, NY). The annexin V-FITC/propidium iodide (AV-FITC/PI) apoptosis detection kit was purchased from Miltenyi Biotec (Shanghai, China).

The pMT3 p38 plasmid was a gift from John Kyriakis (Addgene plasmid # 12658). The MDR1 plasmid was purchased from Genscript (Nanjing, China). The proteasome inhibitor SB203580 and SP600125 were purchased from MedChem Express (MCE, Shanghai, China). The primary antibodies for P-gp, p38, p-p38, p-JNK, p-ERK, PARP, cleaved-PARP, Caspase-9, cleaved-Caspase-9, Caspase-3, cleaved-Caspase-3 and GAPDH were purchased from Cell Signaling Technology (CST, Danvers, MA). Goat anti-rabbit and goat anti-rabbit IgG Fab2 Alexa Fluor (R) antibodies (CST) were used as secondary antibodies. TTM was reserved in DMSO at the concentration of 50 mM. CCK-8 solution was purchased from qcbio Science & Technologies (Shanghai, China)

### Cell lines and cell culture

Parental sensitive human breast cancer cell line MCF-7 and multidrug resistant cell line MCF-7/MDR obtained by the Institute of Hematology and Blood Diseases Hospital (Tianjin, China) were used as an experimental model. Human leukemia cell line K562 and its Doxorubicin-selected P-gp-overexpressing K562/MDR cells were purchased from the KeyGEN BioTECH (Nanjing, China). Both, sensitive and resistant cells were grown in RPMI-1640 medium supplemented with 10% fetal bovine serum at 37 °C with 5% CO_2_ incubator. DOX (1.0 μM) was added to the culture medium to maintain the MDR characteristics of the MCF-7/MDR cells and K562/MDR cells [64].

### Cell viability assays

### MTT assay

Cell proliferation was assessed by MTT colorimetric assay. Briefly, MCF-7, MCF-7/MDR and MCF-10A cells were seeded in 96-well plates at a density of 5 × 10^3^ cells per well and incubated over-night to permit cell adhesion to the cell dishes. Then, the cells were treated with various concentrations of DOX, DNR, EPI, Doc and DDP in the presence or absence of TTM or Ver for 48 hr. 20 μL MTT solution was then added to each well followed incubation for an additional 4 hr. Finally, the purple formazan crystals formed were dissolved in 150 μL of DMSO by gently shaking for 10 mins. The absorbance was evaluated at a test wavelength of 570 nm, and a reference wavelength of 630 nm by ELISA reader (Spectra Max Plus384; Molecular Devices, Sunnyvale, CA). The concentration required to inhibit cell growth by 50% (IC_50_) was calculated by using GraphPad Prism 5.0. Cell viability was calculated using the following formula:

Cell viability = (At/As) × 100%

At and As represent the absorbance of the test substances and solvent control, respectively [65].

The resistance index (RI) was calculated using the following formula: [28, 66] resistance index (RI) = IC_50_ (MDR cells) / IC_50_ (their parental sensitive cells). The reversal fold change, in terms of potency of reversal, was calculated using the following formula: reversal fold change (RF) = IC_50_ (MDR cells) / IC_50_ (MDR cells combined with TTM or Ver treatment).

To measure the inhibitory effect of TTM on p38-MAPK signaling, MCF-7 or MCF-7/MDR cells were treated with serial dilutions of Doc with or without 10 μM SB203580 or 30 μM TTM and/or 10 μM SP600125 for 48 hr, followed by the MTT assay.

All experiments were conducted in triplicate and repeated more than three times. Ver was used as a positive control.

### CCK-8 assay

K562 and K562/MDR cells were seeded in 96-well plates with a density of 5 × 10^3^ cells/well in RPMI-1640 containing 10% FBS. Then the cells were separately exposed to a series of concentrations of compounds complexes for 48 hr and were added to a volume of 100 μL. After 48 hr incubation, 10 μL of the CCK-8 was dropped into each well to culture for 4 hr. The cell viabilities were monitored through measurement of the absorbance at 450 nm on an ELISA reader. The stimulation index (SI) was calculated based on the following formula [67]: SI = (OD_tc_-OD_bl_)/ (OD_ntc_-OD_bl_). (OD_tc_ is the mean OD of the treated cultures, OD_ntc_ is the mean OD of the untreated cultures, and OD_bl_ is the mean OD of the blank wells).

To measure the inhibitory effect of TTM on p38-MAPK signaling, K562 or K562/MDR cells were treated with serial dilutions of Doc with or without 10 μM SB203580 or 30 μM TTM and/or 10 μM SP600125 for 48 hr, followed by the CCK-8 assay.

All experiments were conducted in triplicate and repeated more than three times. Ver was used as a positive control.

### Intracellular accumulation and efflux of DOX assay

The DOX accumulation analyses were performed by fluorescence-activated cell sorter (FACS) analysis and fluorescence microscopy analysis as described previously [68]. In the accumulation assay, both MDR cells and their parent cells were pretreated with or without 10 μM TTM or 10 μM Ver for 4 hr and then incubated with 10 μM DOX for 3 hr. Ver (10 μM) was used as a positive control. In the efflux study, sensitive cells and resistance cells were first cultured with medium containing 10 μM DOX at 37°C for 3 hr, washed 3 times with PBS, then incubated in the absence or presence of 10 μM TTM at 37°C for another 4 hr (or with 10 μM Ver as a positive control). After incubation, all cells were washed twice with ice-cold PBS and subjected to BD Accuri C6 flow cytometry (Becton & Dickinson Company, Franklin Lakes, NJ) to detect the fluorescence produced by DOX for efflux analysis.

### Rh123 flow cytometry assay

The function of P-gp was evaluated by examining the intracellular accumulation and efflux of Rh123 as previously described [68, 69]. In the accumulation analysis, MDR cells and their parent cells were treated with or without 10, 30 or 50 μM TTM or 10 μM Ver for 1.5 hr and then incubated with 5 μM Rh123 at 37 °C for 1.5 hr. In the efflux study, MDR cells and their parent cells were first cultured with medium containing 5 μM Rh123 in the dark for 1.5 hr, washed 2 times with PBS, then incubated in the absence or presence of 10, 30 or 50 μM TTM at 37 °C for additional 1.5 hr (or with 10 μM Ver as a positive control). Subsequently, the culture medium was removed and cells were washed two times with PBS. Finally, the cells were suspended into 250 μL PBS followed by FACS to examine green fluorescence produced.

### The laser confocal to examine the accumulation of Rh123 on MDR cells

To further visualize the effect of TTM on the intracellular retention of Rh123, 5 × 10^3^ cells per well were seeded in the 96-well plate for overnight. The cells were treated with TTM (10, 30 μM) for 48 hr, and were then incubated with 10 μM Rh123 alone or 10 μM Rh123 combination with TTM in the fresh RPMI 1640 medium for 1.5 hr in darkness at 37 °C. After that cells were washed 3 times with cold PBS and images were acquired by the laser confocal.

### Apoptosis assay

Apoptosis was evaluated by FACS consistent with the manufacturer's instructions. Sensitive cells and MDR cells were treated with (+) or without (-) 30 μM TTM combined with 1.0 μM Doc for 48 hr. Cells were then washed twice with cold PBS, suspended in 100 μL of binding buffer at a concentration of 1 × 10^6^ cells/mL. After that, cells were incubated with fluorescein isothicocyannte (FITC)-conjugated annexin V reagent and PI in binging buffer for 30 mins at room temperature as described by the manufacture and finally determined by FACS.

### Colony formation assays

MCF-7 and MCF-7/MDR cells were plated on 6-well plates at a density of eight hundred cells per well. Following incubation overnight, the cells were treated with TTM (10 μM) with or without Doc. The medium was replaced after 48 hr of incubation, and the colonies were further observed 15 days later. The plates were then stained with crystal violet solution (Sigma-Aldrich, St. Louis, MO), and the photographs of the colonies were taken manually [70].

### Plasmid transfection assay

Plasmid preparation was performed and as previously described with some modifications [71, 72]. MCF-7 cells and K562 cells were washed twice with Opti-MEM (Life Technologies, Grand Island, NY). Cells were then transfected with plasmid using Opti-MEM containing 10 μg plasmids in 100 μL of Opti-MEM and Super Electroporator NEPA21 system (NEPAGENE, Japan). After that cells were plated on 6-well plates at a density of 1 × 10^6^ cells cells per well. Twenty-four hours after transfection, cells were treated with 30 μM TTM for 48 hr prior to harvesting.

### Overexpression of P-gp and p38 in MCF-7 and K562 cells

Transfection was performed using the Super Electroporator NEPA21 system. In brief, 1 × 10^6^ cells were transfected using Opti-MEM containing 10 μg plasmid. MCF-7 cells and K562 cells were transfected with the MDR1 plasmid, p38 plasmid or their mock plasmids for 24 hr, and treated with or without 30 μM TTM for another 48 hr, followed by Western blot analysis.

### Quantitative real-time RT-PCR

The qRT-PCR was performed as previously described [73]. The Δ cycle threshold method was used for the calculation of relative differences in mRNA abundance with a LightCycler 480 (Roche Molecular Biochemicals, Mannheim, Germany). Data were normalized to the expression of GAPDH. The results were expressed as fold-changes. The normalized value of the target mRNA of the control group is arbitrarily presented as 1. The sequences of primers used were as follows: MDR1, 5'-CAGAGTCAAGGAGCATGGCA-3' (sense) and 5'-TCAGAGTTCACTGGCGTTT-3' (antisense); MRP1, 5'-ATGGCTCCGACCCGCT-3' (sense) and 5'-AGAGGTAAAAACAAGGCACCCA-3' (antisense); BCRP, 5'-GCACATGCTTGGTGGTCTTG-3' (sense) and 5'-GGCTCAGGATCTCAGGATGC-3' (antisense); and GAPDH, 5'-GAAAGCCTGCCGGTGACTAA-3' (sense) and 5'-AGGAAAAGCATCACCCGGAG-3' (antisense).

### Western blot analysis

Western blot analysis was performed as previously described [72]. MDR cells and their corresponding parental cells were incubated with various concentrations of TTM or 0.1% DMSO for 48 hr. Harvesting after trypsinisation, cells were treated with 1 × RIPA lysis buffer (50 mM Tris–HCl, pH 7.4, 150 mM NaCl, 0.25% deoxycholic acid, 1% NP-40, 1 mM EDTA and protease inhibitors) (Amresco, Solon, USA) to extract the total proteins. An aliquot of proteins from the total cell lysates (30 to 40 μg/lane) was separated by sodium dodecyl sulfate (8%, 12% or 15%) polyacrylamide gelelectrophoresis (SDS–PAGE, BioRad Laboratories, Hercules, CA), wet-transferred to NC membrane (BioRad Laboratories, Hercules, CA) and blotted with primary antibodies specific for Cleaved Caspase-3, Caspase-3, Caspase-9, Cleaved Caspase-9, PARP, Cleaved PARP, P-gp, Phospho-p44/42 MAPK, Phospho-p38 MAPK, Phospho-SAPK/JNK, p38 MAP Kinase, GAPDH, probed with secondary isotype specific antibodies tagged with horseradish peroxidase (Cell Signaling Technology). Bound immuno-complexes were detected using ChemiDOC™ XRS+system (BioRad Laboratories, Hercules, CA).

### The laser confocal to detect the levels of P-gp on the cell membrane

Immunofluorescence was done as previously described before [74]. MCF-7 cells and MCF-7/MDR were cultured on a 96 wells glass culture plate. Cells were treated with TTM for 48 hr. The cells were fixed with warm 4% paraformaldehyde for 15 mins at room temperature. Then abandon the fixed liquid, washed 2 times with PBS for each 5mins and blocked with 5% BSA for 1 hr. Incubation with primary antibodies against P-gp was performed overnight at 4 °C. After incubation, washed 2 times with PBS for each 5 mins. The cells were then incubated with Alexa Flour 488-conjugated secondary antibody for 2 hr at room temperature in the dark. After incubation, washed 2 times with PBS for each 5 mins. The nuclei were stained with DAPI (Beyotime, Haimen, China) for 10 mins before imaging. Images were taken by ImageXpress^®^Micro Confocal (Molecular Devices, USA).

### Statistical analysis

For all experiments, quantitative results were reported as “mean ± SEM” values from at least three experiments performed in a parallel manner. Statistical analysis was performed with the GraphPad Prism 5 software. One-way ANOVA was used to compare each parameter. Next, unpaired, two tailed student's *t* test was performed to identify which group differences accounted for significant overall ANOVA results. ^*^ denotes *P* < 0.05, ^**^ or ^##^ denotes *P* < 0.01 and ^*** or ###^ denotes *P* < 0.001 was considered as significant.

## SUPPLEMENTARY MATERIALS FIGURES



## Abbreviations

ABC: ATP-binding cassette
AV-FITC/PI: annexin V-FITC/propidium iodide
BSA: bovine serum albumin
CST: Cell Signaling Technology
DDP: cisplatin
DMSO: dimethylsulfoxide
DNR: daunorubicin
Doc: docetaxel
DOX: doxorubicin
EPI: epirubicin
FACS: fluorescence-activated cell sorter
FBS: fetal bovine serum
FITC: fluorescein isothicocyannte
MDR: multidrug resistance
P-gp: P-glycoprotein
qRT-PCR: quantitative real-time polymerase chain reaction
RF: reversal fold change
Rh123: rhodamine 123
RI: resistance index
SDS–PAGE: sodium dodecyl sulfate polyacrylamide gelelectrophoresis
SI: stimulation index
TTM: tomentodione M
Ver: verapamil
