# Supplementary material for: Tomentodione M sensitizes multidrug resistant cancer cells by decreasing P-glycoprotein via inhibition of p38 MAPK signaling
Source: Oncotarget. 2017 Oct 19;8(60):101965–83. doi: 10.18632/oncotarget.21949 (PMC5731928; doi:10.18632/oncotarget.21949)
Supplement: Supplementary file 1 [file oncotarget-08-101965-s001.pdf]

Tomentodione M sensitizes multidrug resistant cancer cells by decreasing P-glycoprotein via inhibition of p38 MAPK signaling

SUPPLEMENTARY MATERIALS

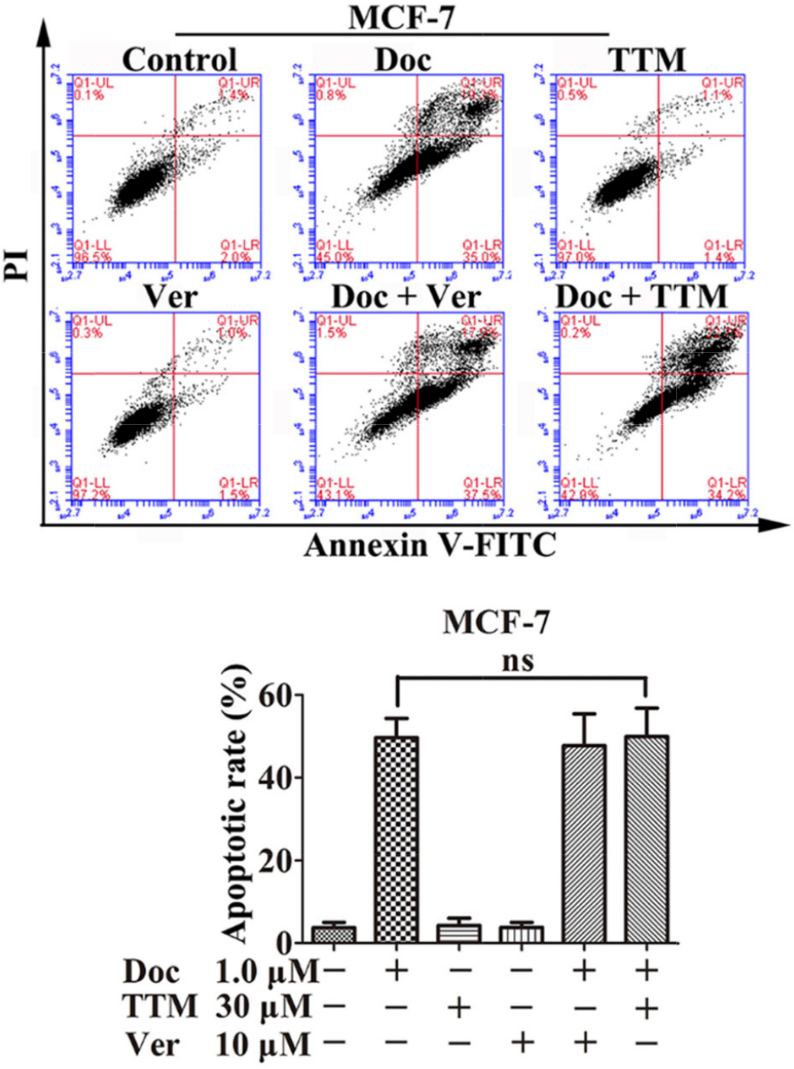

Supplementary Figure 1: Apoptosis in MCF-7 cells. Apoptosis was analyzed by flow cytometry as the percentage of cells labeled by AV-FITC/PI. A representative set of data from three independent experiments is shown.

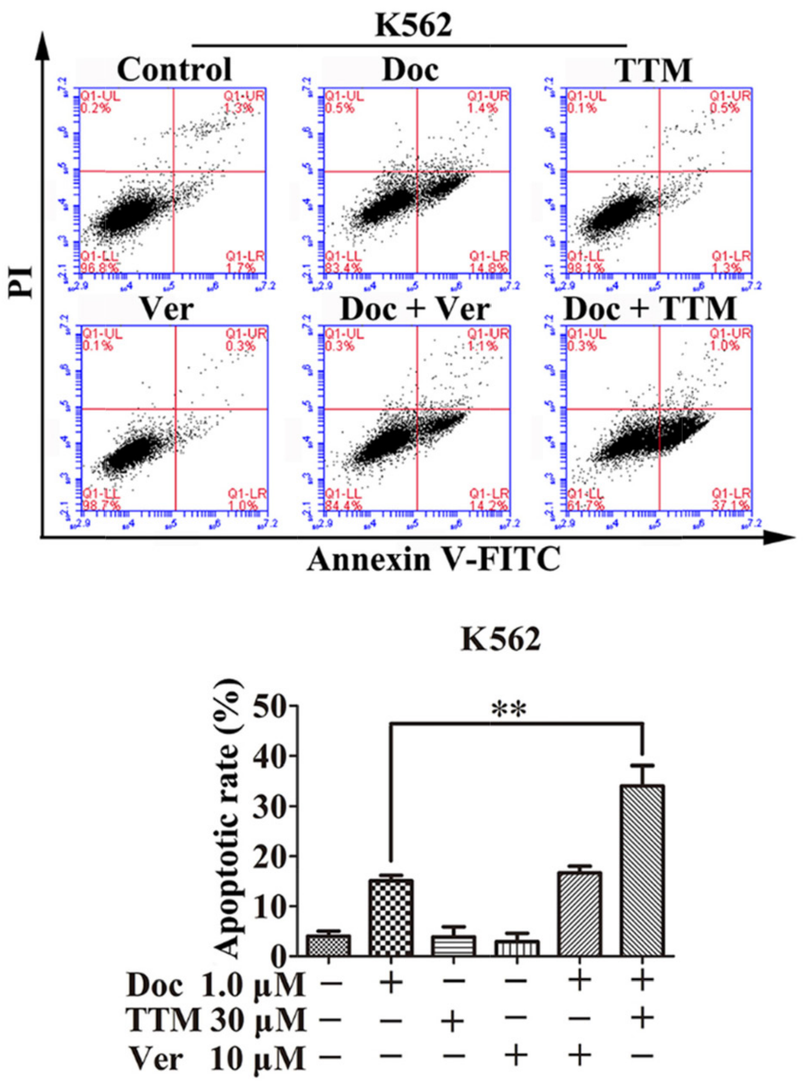

Supplementary Figure 2: Apoptosis in K562 cells. Apoptosis was analyzed by flow cytometry as the percentage of cells labeled by AV-FITC/PI. A representative set of data from three independent experiments is shown. ** denotes $P < 0.01$ compared with Doc single treatment.

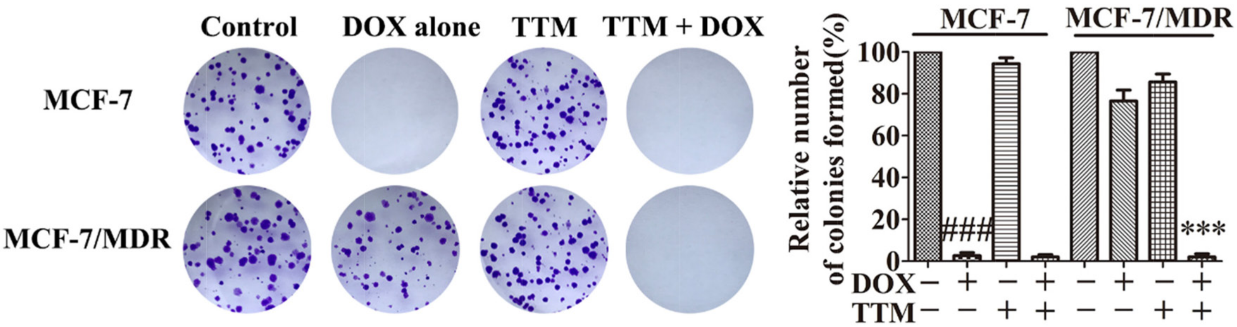

Supplementary Figure 3: The effect of TTM on the clonogenicity of MCF-7/MDR and MCF-7 cells for 48 hr. Colony formation assay of MCF-7 and MCF-7/MDR cells treated with DOX (1.0 μM) in the absence or presence of TTM (30 μM). Cells were re-suspended and plated in 6-well plates containing 1640 plus 10% FBS at a density of 1000 cells per well. Colonies were counted after 15 days. The data are presented as the "mean ± SEM" from three independent experiments. ### denotes $P < 0.001$ versus control. *** denotes $P < 0.001$ compared with Doc single treatment.

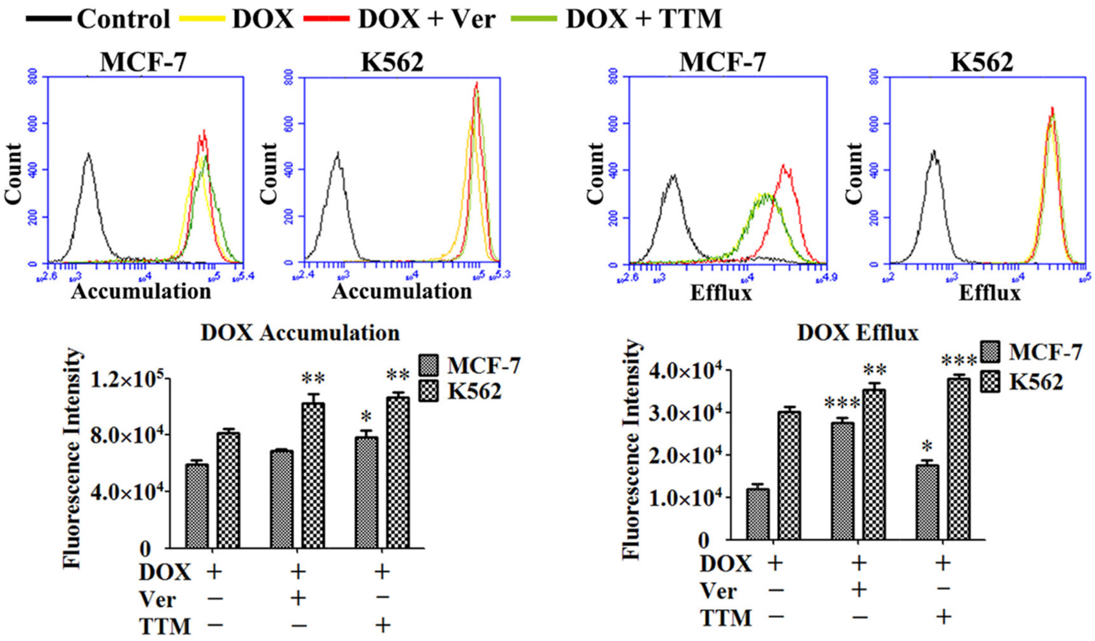

Supplementary Figure 4: In the DOX accumulation assay, MCF-7 or K562 cells were incubated for 4 hr in a medium containing with or without 10 μM TTM or 10 μM Ver, and then washed with warm culture medium for three times. DOX (10 μM) was added for additional 3 hr, washed three times with PBS and examined for the fluorescence of intracellular DOX. In the DOX retention assay, MCF-7 or K562 cells were incubated for 3 hr in a medium containing 10 μM DOX, and then washed with warm culture medium for three times. The cells were incubated for another 4 hr in a medium with or without 10 μM TTM or 10 μM Ver. Subsequently, the cells were washed three times with PBS and examined for the fluorescence of intracellular DOX.* denotes $P < 0.05$, ** denotes $P < 0.01$ and *** denotes $P < 0.001$ versus DOX single treatment.

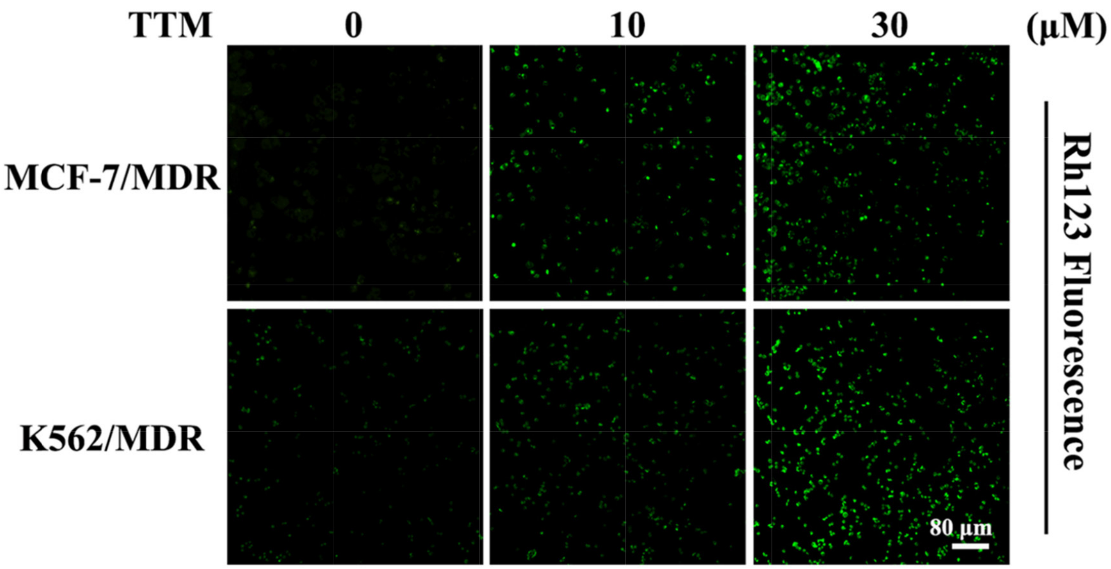

Supplementary Figure 5: The laser confocal detection of Rh123 accumulation in MDR cells after TTM treatment. Cells were treated with 10 or 30 μM TTM for 48 hr, and then Rh123 at 10 μM was added and incubated for an additional 1.5 hr in the dark. Images were acquired digitally by confocal microscope. Bar equals to 80 μm.

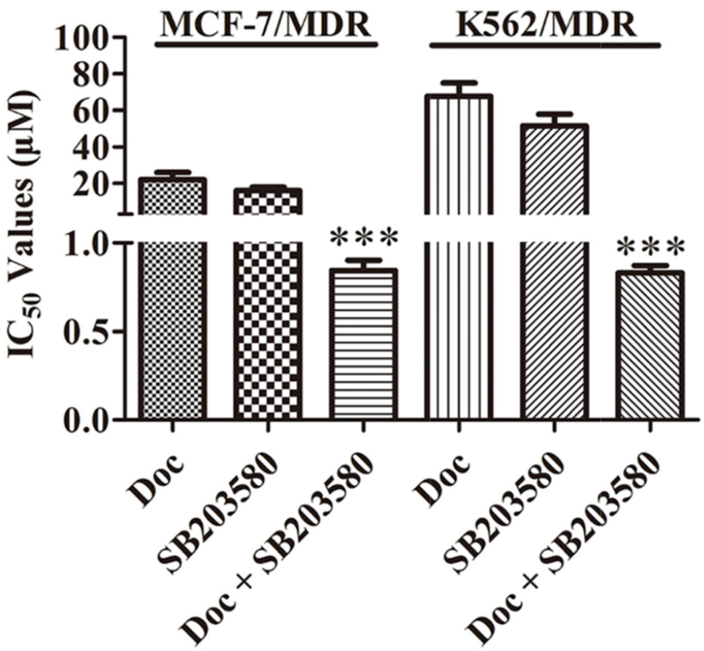

Supplementary Figure 6: MTT and CCK-8 assay showed that SB203580 reversed Doc-resistance in MCF-7/MDR and K562/MDR cells respectively. Cells were treated with the serial dilutions of Doc or SB203580 for 48 hr, or pretreated with 10 μM SB203580 for 1 hr, then treated with the serial dilutions of Doc for 48 hr. *** denotes $P < 0.001$ versus Doc single treatment

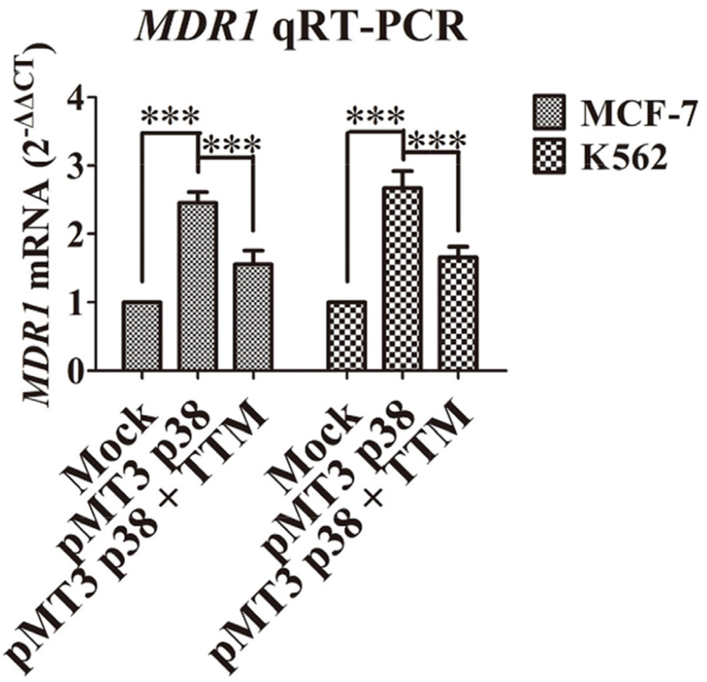

Supplementary Figure 7: MCF-7 or K562 cells were treated with (+) or without (-) 10 µg/L plasmid p38 for 24 hr to increase p38 expression and then were treated with (+) or without (-) TTM for an additional 48 hr. *MDR1* mRNA levels were detected by qRT-PCR. The Δ cycle threshold method was used for the calculation of relative differences in mRNA abundance. Data were normalized to the expression of GAPDH. The results of real-time RT-PCR were expressed as fold-changes. The normalized value of the target mRNA of the control group is arbitrarily presented as 1 (n=6). *** denotes $P < 0.001$ compared with the pMT3 p38 group.
